# Editorial: Machine Learning in Clinical Decision-Making

**DOI:** 10.3389/fdgth.2021.784495

**Published:** 2021-11-18

**Authors:** Amanda C. Filiberto, Ira L. Leeds, Tyler J. Loftus

**Affiliations:** ^1^Department of Surgery, University of Florida, Gainesville, FL, United States; ^2^Department of Surgery, Yale School of Medicine, New Haven, CT, United States

**Keywords:** artificial intelligence, machine learning, reinforcement learning, decision analysis, decision-making

The “Machine Learning in Clinical Decision-Making” Research Topic assimilates evidence and perspectives from researchers and thought leaders that are pursuing the safe, effective development, and clinical application of machine learning systems to augment clinical decision-making across a wide array of specialties including cardiology, neurology, audiology, intensive care, and oncology. This editorial summarizes key points from the Research Topic.

Electronic health record (EHR) systems have become widespread amongst health care systems globally. The resulting EHR databases have generated large, heterogeneous datasets that offer new opportunities to design and implement smarter health care systems by minimizing manual data entry, introducing objectivity where hypothetical-deductive reasoning fails, and providing accurate predictions and classifications that tailor care to individual patients' needs. Despite the promising role for artificial intelligence (AI) techniques and technologies to improve patient care, several substantial barriers to clinical adoption remain.

Many ethical dilemmas surround the use of AI in healthcare. Machine learning algorithms trained to optimize a certain endpoint may make medically sound recommendations without reflecting a patient's ultimate goals of care, which often differ from textbook medical outcomes. Additionally, algorithms trained on biased data sets may produce biased outputs, which may be detrimental if the target population demographics and other characteristics are misaligned. There is also a lack of transparency regarding how many AI algorithms arrive at predictions, which has important implications for care delivery. Currently, despite impressive efficacy in retrospective and observational studies, there is limited level I evidence supporting the use of health care AI for decision support, suggesting a need for more high-level evidence, especially randomized trials.

Electrocardiography (ECG) is an efficient, easily accessible, and commonly performed method for screening and diagnosing cardiovascular disease, a major contributor to potentially preventable mortality and morbidity. Among cardiovascular diseases, heart failure is particularly difficult to recognize due to heterogeneity of underlying pathology and clinical manifestations. Grun et al. demonstrate the ability of AI to accurately predict heart failure from standard 12-lead ECGs, highlighting the potential for AI to promote early diagnosis and treatment using routine clinical data.

Electroencephalography (EEG) is used in the diagnosis, monitoring, and prognostication of many neurological ailments including seizure, coma, sleep disorders, brain injury, and behavioral abnormalities. Similar to cardiovascular disease, these neurologic diseases are heterogenous and often present with diagnostic uncertainty. Saba-Sadiya et al. propose a flexible, unsupervised model that applies to novel EEG data for a variety of clinical decision tasks, including coma prognostication and neurodegenerative illness detection. This work represents an important foundation for future investigations. Epilepsy affects 50 million people worldwide; approximately one third of all cases are refractory to medications. If a discrete cerebral focus is identified, then neurosurgical resection can be curative. Alim-Marvasti et al. provide evidence that machine learning models trained on a combination of chronological clinical seizure manifestations and an imaging feature can enhance epileptogenic lobe localization, which is a necessary step in achieving optimal surgical outcomes for medication-refractory epilepsy.

In audiology, large amounts of patients' data are measured but are distributed primarily over local clinical databases with unique structures, data elements, and variable names, which hinders external validation and multi-center investigations. Saak et al. illustrated the feasibility of automatically predicting common audiological functional parameters from audiological measures using separate lasso regression, elastic net, and random forest algorithms, which had similar, strong predictive performance. The trained models underlie a prototype for a broadly applicable audiology clinical decision-support system that would function well across local clinical databases despite their unique structures, data elements, and variable names.

Intensive care units (ICUs) serve critically ill patients who require near-continuous surveillance or advanced organ support. Medication dosing can be challenging for critically ill patients because they are often affected by gastrointestinal, hepatic, and renal dysfunction, which affect medication absorption, metabolism, and excretion. Several data-driven medication dosing models have been proposed but have limited ability to assess inter-individual differences and compute individualized doses. Eghbali et al. developed a sedation management agent using deep reinforcement learning which was associated with improved ICU blood pressure management compared with clinicians' performance. The framework proposed by the authors holds promise for automating dosing for other medications commonly used in ICUs.

Smartphones, wearables, and other devices providing medically relevant information generated directly by individuals outside the healthcare system are an emerging trend and can augment existing EHR data for model training purposes. Kyriazakos et al. describe how physiological, psychological, social, and environmental biomarkers can be used to train machine learning algorithms to determine the quality of life of cervical cancer patients and identify novel treatments. Shickel et al. explored the benefits of incorporating novel measurements from wrist-worn activity sensors into EHR data and using resulting datasets and temporal deep learning models to predict patients' illness severity. These results demonstrate the power of non-traditional patient data for making predictions and classifications that have the potential to enhance clinical decision-making.

The editors hope you enjoy the “Machine Learning in Clinical Decision-Making” Research Topic and that your clinical and research efforts in this realm are enriched by the evidence and wisdom shared by the contributing authors.

## Author Contributions

ACF, ILL, and TJL made substantial contributions to the conception and interpretation of data for the work, provided approval for publication of the content, and agreed to be accountable for all aspects of the work. ACF drafted the manuscript. ILL and TJL provided critical revisions. All authors contributed to the article and approved the submitted version.

## Funding

TJL was supported by the National Institute of General Medical Sciences of the National Institutes of Health under Award Number K23 GM140268.

## Author Disclaimer

The content is solely the responsibility of the authors and does not necessarily represent the official views of the National Institutes of Health.

## Conflict of Interest

The authors declare that the research was conducted in the absence of any commercial or financial relationships that could be construed as a potential conflict of interest.

## Publisher's Note

All claims expressed in this article are solely those of the authors and do not necessarily represent those of their affiliated organizations, or those of the publisher, the editors and the reviewers. Any product that may be evaluated in this article, or claim that may be made by its manufacturer, is not guaranteed or endorsed by the publisher.

